# Supplementary material for: Accounting for drinking water quality in measuring multidimensional poverty in Ethiopia
Source: PLoS One. 2020 Dec 15;15(12):e0243921. doi: 10.1371/journal.pone.0243921 (PMC7737968; doi:10.1371/journal.pone.0243921)
Supplement: S2 Fig — (DOCX) [file pone.0243921.s002.docx]

|  |
| --- |
|  |
| 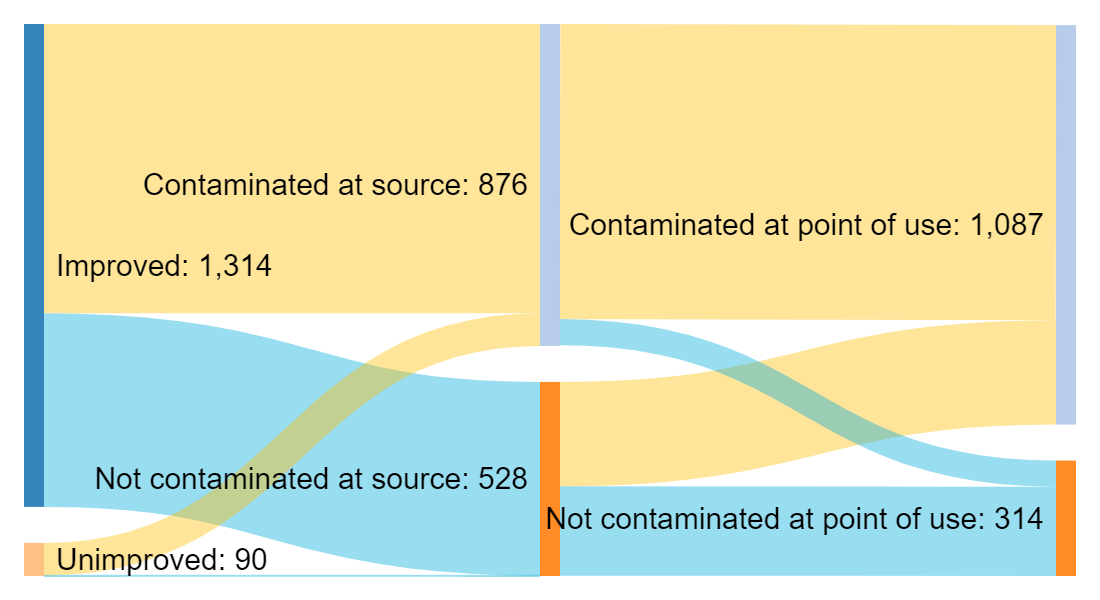 |

**S2 Figure. Improved and unimproved water source types and their *E. coli* contamination status at source and point of use in urban areas.**
