# Supplementary material for: Accounting for drinking water quality in measuring multidimensional poverty in Ethiopia
Source: PLoS One. 2020 Dec 15;15(12):e0243921. doi: 10.1371/journal.pone.0243921 (PMC7737968; doi:10.1371/journal.pone.0243921)
Supplement: S1 Table — (DOCX) [file pone.0243921.s003.docx]

**S1 Table. Summary of MPI indicator for three scenarios.**

| Dimensions & Indicators | Weight | Deprived if... | | |
| --- | --- | --- | --- | --- |
|  |  | No improved source | No safely managed source | No safely managed and free from contamination at point of use |
| **Education** |  |  |  |  |
| Child school attendance | 0.17 | 20.0% | 20.0% | 20.0% |
| Years of schooling | 0.17 | 47.4% | 47.4% | 47.4% |
| **Health** |  |  |  |  |
| Nutrition | 0.11 | 18.1% | 18.1% | 18.1% |
| Drinking water | 0.11 | 13.2% | 92.3% | 97.4% |
| Sanitation | 0.11 | 26.7% | 26.7% | 26.7% |
| **Living Standards** |  |  |  |  |
| Electricity | 0.08 | 69.5% | 69.5% | 69.5% |
| Cooking fuel | 0.08 | 92.6% | 92.6% | 92.6% |
| Housing | 0.08 | 85.0% | 85.0% | 85.0% |
| Assets | 0.08 | 51.2% | 51.2% | 51.2% |

Notes: Author’s calculations based on ESS 2016. Number of observations (households)=4,464.
