# Supplementary material for: Accounting for drinking water quality in measuring multidimensional poverty in Ethiopia
Source: PLoS One. 2020 Dec 15;15(12):e0243921. doi: 10.1371/journal.pone.0243921 (PMC7737968; doi:10.1371/journal.pone.0243921)
Supplement: S2 Table — (DOCX) [file pone.0243921.s004.docx]

**S2 Table.** **MPI results for three scenarios by region and type of residence**

|  |  | No improved source | | | No safely managed source | | | No safely managed and free from contamination at point of use | | |
| --- | --- | --- | --- | --- | --- | --- | --- | --- | --- | --- |
|  | N | MPI | H | A | MPI | H | A | MPI | H | A |
| National | 4,464 | 0.369 | 68.8% | 53.6% | 0.474 | 81.1% | 58.5% | 0.480 | 82.2% | 58.4% |
| Rural | 2,993 | 0.457 | 83.7% | 4.7% | 0.571 | 94.8% | 60.2% | 0.574 | 95.2% | 60.3% |
| Small towns | 366 | 0.149 | 32.0% | 46.5% | 0.251 | 51.1% | 49.1% | 0.267 | 55.0% | 48.5% |
| Medium & Large towns | 1,105 | 0.099 | 23.6% | 42.0% | 0.175 | 38.2% | 45.7% | 0.189 | 41.5% | 45.7% |
|  |  |  |  |  |  |  |  |  |  |  |
| Addis Ababa | 203 | 0.0249 | 5.7% | 41.0% | 0.039 | 9.2% | 42.4% | 0.043 | 9.6% | 44.3% |
| Amhara | 906 | 0.419 | 76.7% | 54.7% | 0.530 | 89.2% | 59.4% | 0.534 | 89.8% | 59.4% |
| Oromia | 913 | 0.384 | 71.7% | 53.6% | 0.493 | 84.0% | 58.7% | 0.501 | 85.8% | 58.4% |
| SNNP^a^ | 1,028 | 0.351 | 67.7% | 51.9% | 0.468 | 82.8% | 56.4% | 0.469 | 83.1% | 56.4% |
| Tigray | 531 | 0.339 | 65.3% | 52.0% | 0.437 | 75.8% | 57.6% | 0.442 | 77.0% | 57.5% |
| All Others | 883 | 0.380 | 67.7% | 56.1% | 0.482 | 78.1% | 61.7% | 0.491 | 80.4% | 61.1% |

Notes: Authors’ calculation based on ESS 2016. Notations are: N= Number of observations(households) in the sample , MPI= adjusted multidimensional headcount, H = multidimensional headcount or incidence of poverty, and A = average intensity of poverty.

^a^Southern Nations, Nationalities, and Peoples' Region
